# Evaluation of Postoperative Pain When Adding a Tibial Nerve Block to the Femoral Nerve Block for Total Knee Arthroplasty

**DOI:** 10.3390/jcm13154387

**Published:** 2024-07-26

**Authors:** Alejandra Mira-Puerto, Pedro Romero-Aroca, Alfredo Rodríguez-Gangoso, Albert Ferrando-de Jorge, Mireia Duart-Oltra, Pilar Sala-Francino, Mari Carmen Martínez-Segovia, Marc Baget-Bernaldiz

**Affiliations:** 1 Anaesthetic Service, Hospital Universitat Sant Joan, Institut d’Investigació Sanitària Pere Virgili [IISPV], Universitat Rovira i Virgili, 43204 Reus, Spain; mireiaduart@gmail.com (M.D.-O.); psalafrancino@gmail.com (P.S.-F.); 2 Ophthalmology Service, Hospital Universitat Sant Joan, Institut d’Investigació Sanitària Pere Virgili [IISPV], Universitat Rovira i Virgili, 43204 Reus, Spain; romeropere@gmail.com (P.R.-A.); mbaget@gmail.com (M.B.-B.); 3 Traumatology Service, Hospital Universitat Sant Joan, Institut d’Investigació Sanitària Pere Virgili [IISPV], Universitat Rovira i Virgili, 43204 Reus, Spain; doctor.alfredo.rodriguez@gmail.com (A.R.-G.); aferrandoj@gmail.com (A.F.-d.J.); 4 Anaesthetic Service, Hospital Universitario Virgen de la Arrixaca, 30120 Murcia, Spain; marimartinezsegovia@gmail.com

**Keywords:** total knee arthroplasty, femoral nerve block, tibial nerve block, opioids, postoperative analgesia

## Abstract

**Background**: The aim of this study was to compare the postoperative analgesic efficacy when a tibial nerve block was added to the femoral nerve block for total knee arthroplasty (TKA). **Methods**: A total of 60 patients were randomly assigned to the experimental group (EG) or the control group (CG) in a 1:1 ratio. The thirty patients who formed the CG underwent an ultrasound-guided femoral nerve block together with neuraxial anaesthesia and the administration of opioids and NSAIDs through an intravenous elastomeric pump for the management of the postoperative pain; the other thirty, who formed the EG, underwent neuraxial anaesthesia together with femoral and tibial nerve blocks. The efficacy of the analgesic effect was evaluated based on the numerical pain rating scale (NPRS) and on the need for analgesic rescue at different time intervals within 48 h after surgery. **Results**: At 24 h, the mean NPRS score in the EG and CG at rest was 1.50 ± 1.19 and 1.63 ± 1.60 [*U* = 443.5, *p* = 0.113], respectively. With joint movement, the mean NPRS score was 2.80 ± 1.49 and 3.57 ± 1.79 [*U* = 345, *p* = 0.113], respectively. Ten patients in the EG [33.3%] and 24 in the CG [80%] required rescue analgesia [Phi = 0.471, *p* < 0.001]. At 48 h, the mean NPRS score in the EG and CG at rest was 0.33 ± 0.60 and 0.43 ± 0.72 [*U* = 428, *p* = 0.681], respectively. With movement, the mean NPRS score was 1.03 ± 0.99 in the EG and 1.60 ± 1.07 in the CG [*U* = 315, *p* = 0.038]. No patient in the EG group required rescue analgesia, while three patients in the CG [10%] did [Phi = 0.229, *p* = 0.076]. The mean opioid dosage in the CG was 300 mg, whereas in the EG it was 40 mg ± 62.14 [*U* < 0.05, *p* < 0.001]. **Conclusions**: Adding a tibial nerve block to the femoral nerve block in TKA may achieve the same analgesic efficacy within 48 h after surgery and would reduce the systematic use of opioids.

## 1. Introduction

Orthopaedic surgery has experienced exponential growth over the last decade, mainly due to the ageing of the population in developed countries. Total knee arthroplasty (TKA) together with hip replacement are currently the two most common surgeries performed.

More than half of the patients undergoing TKA have moderate or severe pain, especially in the immediate postoperative period [1]. Hence, it is still a challenge to manage the pain resulting from this procedure [2].

Pain is a subjective phenomenon that can be defined as an unpleasant sensory and emotional experience associated with actual or potential tissue damage [3]. Postoperative pain generates suffering and drives up healthcare costs since it increases perioperative morbidity, prolongs hospitalisation, and increases opioid consumption and the probability of postoperative chronic pain [4,5]. In addition, it lowers patient satisfaction. Consequently, there is currently great interest in improving the management of pain from TKA and minimising the side effects caused by the opioids used in this surgery [6].

The most common analgesic methods used for pain control after a TKA surgery are epidural anaesthesia, pericapsular infiltration with local anaesthetics, peripheral nerve blocks, and intravenous drugs. All of these methods have some limitations, such as acute urinary retention, vomiting, motor nerve block, delayed rehabilitation, and the masking of nerve injuries.

Peripheral nerve blocks have shown some advantages. They cause fewer postoperative complications, allow the use of opioids to be reduced, enable faster rehabilitation programs, and are cost-effective [7].

The innervation of the knee is very complex as it involves several nerves, such as the femoral, sciatic, and obturator nerves. Broadly speaking, the anterior capsule of the knee is innervated by the femoral nerve, while the posterior capsule is innervated by the sciatic nerve (common peroneal and tibial nerves). A sciatic nerve block lasts longer and can mask peroneal nerve injuries during TKA surgery, which occur in 0.3–10% [8]. The tibial nerve plays an important role in the innervation of the posterior and lateral part of the knee capsule [9].

The objective of this interventional study was to compare postoperative analgesic efficacy in two groups of patients who underwent TKA: the experimental group (EG) underwent neuraxial anaesthesia together with peripheral femoral and tibial nerve blocks, and the control group (CG) underwent neuraxial anaesthesia, a femoral nerve block and elastomeric pump infusion of non-steroidal anti-inflammatory drugs (NSAIDs) and opioids.

## 2. Material and Methods

### 2.1. Setting

We used a sample size of 105 patients eligible to undergo TKA. All patients came from our healthcare area [Hospital Universitari Sant Joan de Reus, Spain].

### 2.2. Study Design

**Figure 1:**
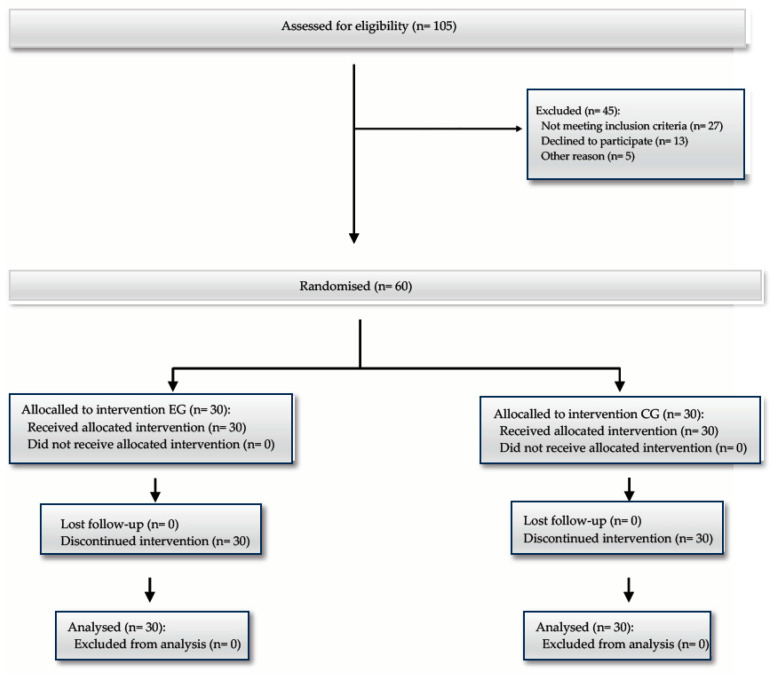
The flow diagram of the study. Of 105 eligible patients, 13 decided not to participate in the study and 27 were excluded due to the presence of systemic diseases that could have altered the assessment of the analgesic effect. Sixty patients were randomly assigned in a 1:1 ratio. No patients were lost during the study period.

A prospective, interventional, randomised clinical trial was conducted from October 2022 to February 2024. One hundred and five patients were screened for eligibility, of whom 45 were excluded. Of those excluded, 27 had systemic diseases that might have interfered with the analgesic effect: 23 had type 2 diabetes mellitus, 2 Parkinson’s disease, and 2 multiple sclerosis. Thirteen patients decided not to participate in the study. Ultimately, 60 patients were enrolled in the study and were randomly assigned in a 1:1 ratio to either the EG or the CG by a resident not affiliated with the study and according to their scheduled surgery date. No patients were lost after randomisation (Figure 1). Consolidated Standards of Reporting Trials (CONSORT) guidelines were considered for study design.

### 2.3. Inclusion and Exclusion Criteria

Inclusion criteria:

▯ Patients eligible for TKA through neuraxial anaesthesia.
▯ Aged 50–80 years. ASA I–III.
▯ BMI <35 kg/m^2^.
▯ Correct understanding of the nature of this study.

Exclusion criteria:

▯ The presence of any systemic disease that may have altered the effect of the analgesia (diabetes mellitus, neuropathy, vasculopathy).
▯ Psychiatric disorder.
▯ History of allergy or significant intolerance to local anaesthetics, paracetamol, NSAIDs, metamizole, and tramadol.
▯ Contraindication for intrathecal anaesthesia:
  - Absolute: patient does not give consent or adequately comply with the procedure. Infection in the infiltration site.
  - Relative: coagulation disorders

### 2.4. Ethics and Consent

The study was carried out with the approval of the local ethics committee (approval no. 13-CEIM:05/2021) on 27 May 2021 and the Spanish and European Medicine Agency (approval EudraCT 2020-005443-23) and in accordance with the revised guidelines of the Declaration of Helsinki. Patients gave signed consent after being informed about the purpose of the study.

### 2.5. Total Knee Arthroplasty Surgery Technique

All surgical procedures were performed by the same pair of orthopaedic surgeons with the patient under regional anaesthesia in the supine position. For the implantation of the prosthesis, a standard surgical protocol was followed. The medial parapatellar approach was employed in all cases to dislocate and denervate the patella. Both cruciate ligaments were excised during the surgery, while preserving both collateral ligaments. Bone resection was carried out sequentially according to the surgical technique, depending on the company design and preoperative planning. Finally, the prosthesis was implanted. Once the spacers were tested both in extension and flexion, and there was an adequate balance of the soft tissue, the final implants were positioned with a previous lavage of the joint.

The two prosthesis designs used were Innex (Zimmer Biomet, Warsaw, IN, USA) and Gemini (Waldemar Link, Hamburg, Germany). All surgeries were performed with the use of a tourniquet until the implantation of the prosthesis. No drainage bottles were used if the surgical incision was closed in the normal way and if there was no excessive bleeding after tourniquet use.

### 2.6. Anaesthetic Perioperative Management in the Experimental and Control Groups

All patients were anaesthetised by the same anaesthesiologist and underwent an ultrasound-guided femoral nerve block under mild sedation with IV midazolam 2 mg thirty minutes before surgery, located using a 13–6 Hz frequency probe. Once the femoral nerve was located lateral to the femoral artery and vein, and after ruling out the intraneural administration of the anaesthetic by using neurostimulation, ropivacaine 90 mg was administered peripherally to the femoral nerve in a single puncture with a 100 mm needle.

In addition, those patients in the EG underwent an ultrasound-guided tibial nerve block in a complementary way. With the patient in a lying position, with the limb in external rotation and the knee flexed, the tibial nerve was located approximately 2 cm under the sciatic nerve bifurcation in the popliteal fossa, where ropivacaine 60 mg was administered in a single puncture.

Finally, all patients underwent intrathecal anaesthesia using 0.5% bupivacaine 11–15 mg at the L3–L4 level. Both groups received prophylaxis with antibiotics along with IV dexamethasone 4 mg and dexketoprofen 50 mg before the incision was made.

### 2.7. Evaluation of Time of Ischemia, Surgery Duration and Blood Loss

The duration of ischemia was measured from the time when the tourniquet was applied around the patient’s thigh until it was removed after component implantation. The duration of surgery was timed from the midline skin incision of the knee to its closure. Finally, the estimated blood loss was quantified by measuring the amount of blood stored in the aspirators and subtracting the saline solutions used to irrigate the wound.

### 2.8. Evaluation of Motor and Sensitive Block after Surgery: Bromage

Patients were assessed for motor and sensory blockade in the post-anaesthesia care unit (PACU) using the Bromage scale. Bromage is a qualitative method that evaluates the degree of motor blockade after anaesthesia in terms of the patient’s ability to move their lower limbs. It comprises the following 4 categories:

▯ Complete: Unable to move feet or knees.
▯ Almost complete: Able to move feet only.
▯ Partial: Just able to move knees.
▯ None: Full flexion of knees and feet.

The time required for patients to regain the full flexion of their knees and feet was measured from the start of spinal anaesthesia.

### 2.9. Postoperative Pain Study

The presence of postoperative pain was assessed at different time intervals within 48 h after the surgery using the numerical pain rating scale (NPRS), in which patients rated their pain from 0 to 10 as follows:

▯ 0: no pain.
▯ 1–3: mild pain.
▯ 4–6: moderate pain.
▯ 7–10: severe pain.

Knee pain was assessed at rest and with movement. Pain with movement was assessed by knee flexion and extension within 24 h of surgery and by standing and walking thereafter.

In addition, the presence of pain was indirectly evaluated through the need for rescue analgesia and the dose of opioids consumed in each group. The variables potentially related to rescue analgesia were studied using logistic regression analysis. The variables potentially linked to the dose of opioids consumed were studied by means of multiple linear regression analysis.

### 2.10. Rescue Analgesia

Patients in the CG received IV 100 mg tramadol and 10 mg metoclopramide at the end of surgery. Once in the PACU, all patients in the CG were connected to a 48 mL intravenous elastomeric pump to administer 200 mg tramadol, 100 mg dexketoprofen, and 10 mg metoclopramide at a continuous flow rate of 2 mL/h. If rescue analgesia was required in the CG, paracetamol 1 g/6 h was administered as the first-line treatment and methimazole 2 g/6 h as the second-line treatment. Rescue analgesia was administered when the NPRS was >3.

Once in the PACU, the EG received 1 g/6 h paracetamol 1 intravenously if analgesia was required. If additional analgesia was needed, 2 g/6 h methimazole was administered as the second-line treatment. Rescue analgesia was administered when the NPRS was >3.

### 2.11. Statistical Analysis

Data in this study were analysed using the SPSS software package (software IBM^®^ SPSS^®^ version 25.0, IBM Corp., Armonk, NY, USA). The dependent variable in this study was the pain after TKA surgery measured directly using the NPRS and indirectly through rescue analgesia and opioid consumption.

At baseline, the independent demographic and clinical variables were gender, age, ASA level, BMI, the Barthel index, the haemoglobin levels, the estimated glomerular filtration rate (eGFR), the thigh diameter, and the anaesthetic technique used. Intraoperatively, the independent variables were the time of surgery, the time of ischemia, the estimated blood loss, the type of prosthesis implanted, and the use of tranexamic acid. Once the surgery was completed, the Bromage score was evaluated.

The sample size was calculated using the GRANMO sample size calculator. Two independent medians were compared; accepting an alpha risk of 0.05 and a beta risk of less than 0.2 in a bilateral contrast, it was found that at least 18 patients were needed in each group to detect a difference equal to or greater than 2 units. The common standard deviation was assumed to be 2, and the rate of loss to follow-up was estimated to be 10%.

For qualitative data, we analysed the frequency and percentage in each category. A descriptive statistical analysis was made of the quantitative data. The normal data curve was evaluated using the Kolmogorov–Smirnov test. Differences between quantitative variables with normal distribution were examined using Student’s *t*-test; otherwise we used the Mann–Whitney *U* test. Inferential analysis for qualitative data was carried out using the chi-squared table and the determination of the Phi test. To correlate two continuous quantitative variables with normal distribution, we used Pearson’s parametric coefficient. For categorical variables we used Spearman’s coefficient. We used logistic regression analysis to study which independent variables were significantly related to the need for rescue analgesia. We used multiple regression analysis to study which independent variables were related significantly to opioid consumption. *p* < 0.05 was considered statistically significant.

## 3. Results

### 3.1. Study of Demographic and Clinical Variables at Baseline

The proportion of men and women was similar in the two groups. The mean age, Barthel index, BMI, haemoglobin level, and thigh diameter were also similar. There were statistically significant differences regarding the ASA level between groups since the EG had more patients with an ASA of 3 [*p* = 0.034] [Table 1].

### 3.2. Study of Intraoperative Variables

We studied the type of prosthesis implanted, the administration of tranexamic acid, the time of surgery, the time of ischemia, and blood loss. There were no statistically significant differences in any of these variables. The results are shown in Table 2.

### 3.3. Bromage

The Bromage score was 243.0 ± 56.59 and 252 ± 50.45 in the EG and CG, respectively [*U* = 388, *p* = 0.351].

### 3.4. Postoperative Pain Study

**Figure 2:**
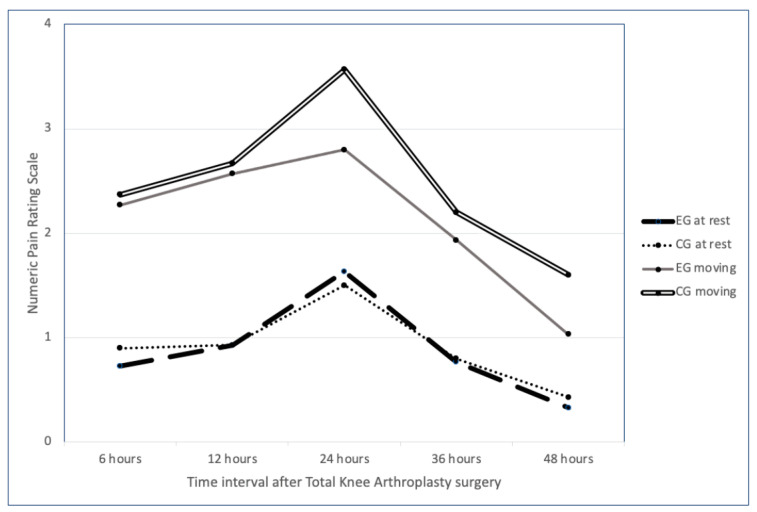
Numerical pain rating scale values shown for both groups in the study. The experimental (EG) and control groups (CG) showed similar mean NPRS values at rest within the first 48 h after total knee arthroplasty surgery. However, with movement, the control group showed higher NPRS values at 24, 36, and 48 h after surgery. The difference was statistically significant at 48 h.

Postoperative pain was assessed at rest and with movement of the knee using the numerical pain rating scale (NPRS) at different time intervals within the first 48 h after surgery in both groups [Figure 2].

**Figure 3:**
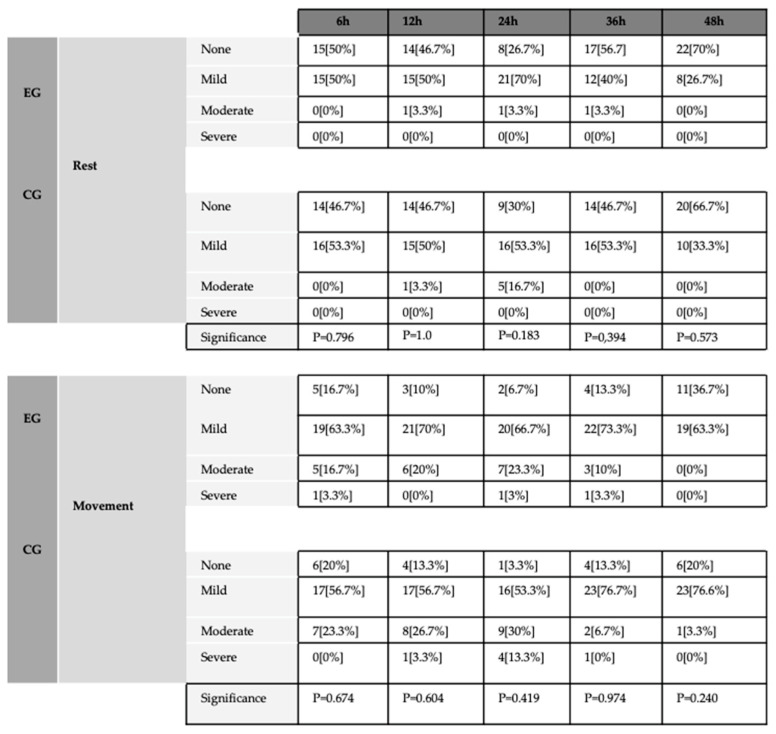
Number of patients who rated their pain as absent, mild, moderate, or severe in the experimental and control groups.

Figure 3 shows the proportion of patients who rated pain as absent, mild, moderate, and severe for each group studied at different time intervals after surgery.

At rest, 3 and 6 patients rated their pain as moderate in the EG and CG, respectively. With joint movement, 21 patients in the EG and 26 in the CG rated their pain as moderate. However, there were no statistically significant differences between the groups.

#### 3.4.1. Pain Evaluation 6 h after Surgery

The mean NPRS score in the EG and in the CG at rest was 0.73 ± 0.86 and 0.90 ± 1.0, respectively [*U*= 427, *p* = 0.713]. The mean NPRS score with movement was 2.27 ± 1.68 and 2.37 ± 1.81 in the EG and CG, respectively [*U* = 439, *p* = 0.864]. Although there were no statistically significant differences between them, nine patients in the EG [34.6%] and 17 in the CG [65.4%] required rescue analgesia; this difference was statistically significant [Phi = 0.269, *p* = 0.037].

#### 3.4.2. Pain Evaluation 12 h after Surgery

The mean NPRS score in the EG and in the CG at rest was 0.97 ± 1.09 and 0.93 ± 1.04, respectively [*U* = 445.5, *p* = 0.943]. The mean NPRS score with movement in the EG was 2.57 ± 1.40, while in the CG it was 2.67 ± 1.76 [*U* = 434, *p* = 0.809].

There were only seven patients in the EG who required rescue analgesia [28%], whereas 18 patients [72%] in the CG required it; this difference was statistically significant [Phi = 0.372, *p* = 0.004].

#### 3.4.3. Pain Evaluation 24 h after Surgery

The mean NPRS score of the EG and CG at rest was 1.50 ± 1.19 and 1.63 ± 1.60, respectively [*U* = 443.5, *p* = 0.113]. With movement, the mean NPRS score in the EG was 2.80 ± 1.49 and 3.57 ± 1.79 in the CG [*U* = 345, *p* = 0.113].

Ten patients in the EG [33.3%] and 24 in the CG [80%] required rescue analgesia; this result was statistically significant [Phi = 0.471, *p* < 0.001].

#### 3.4.4. Pain Evaluation 36 h after Surgery

The mean NPRS score was 0.77 ± 1.16 and 0.80 ± 0.84 in the EG and CG, respectively [*U* = 409, *p* = 0.508]. With movement, the mean NPRS score was 1.93 ± 1.78 and 2.20 ± 1.56 in the EG and CG, respectively [*U* = 375, *p* = 0.255]. There was no difference between the groups regarding rescue analgesia. Only seven patients in the EG [23.3%] and nine in the CG required it [30%] [Phi = 0.075, *p* = 0.559].

#### 3.4.5. Pain Evaluation 48 h after Surgery

The mean NPRS score was 0.33 ± 0.60 and 0.43 ± 0.72 in the EG and CG, respectively [*U* = 428, *p* = 0.681]. With movement, the mean NPRS score was 1.03 ± 0.99 and 1.60 ± 1.07 in the EG and CG, respectively [*U* = 315, *p* = 0.038].

No patient in the EG group required rescue analgesia, while only three patients in the CG [10%] did. [Phi = 0.229, *p* = 0.076].

### 3.5. Study of Rescue Analgesia

Nineteen patients in the EG [63.3%] and 29 in the CG [96.7%] required rescue analgesia during the postoperative period studied [Phi = 0.417, *p* < 0.001]. Patients in the EG required a mean of one rescue analgesia [1.10 ± 1.15], whereas in the CG nearly 2.5 [2.37 ± 1.15] did [*p* < 0.001]. Using a logistic regression analysis, we evaluated the independent variables that could be related to rescue analgesia. The anaesthetic technique was the only variable that was related to rescue analgesia. [Table 3] [X2(8) = 0.6432, *p* = 0.039]. The model explained 39.8% (Nagelkerke R^2^) of the variance regarding the need for rescue analgesia.

#### Probability of Needing Rescue Analgesia

**Figure 4:**
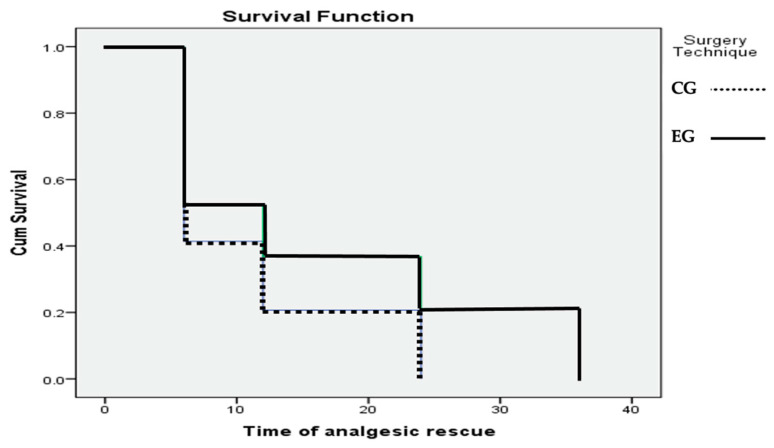
Kaplan–Meier survival curve. The CG was more likely to require rescue analgesia compared to the EG at each time interval studied. A patient in the CG had an 80% and 100% probability of requiring rescue analgesia at 12 and 24 h after surgery, compared with 50% and 60% in the EG.

Using Kaplan–Meier survival curves, we calculated the probability of requiring rescue analgesia within 48 h after TKA surgery [Figure 4]. The probability was greater in the CG at all time intervals studied [Log Rank = 0.355; *p* = 0.04].

### 3.6. Study of the Consumption of Opioids

All patients from the CG took opioids, whereas only 10 from the EG did [33.3%] [Phi = −0.707, *p* < 0.001]. The mean dose of opioids in the CG was 300 mg, whereas in the EG it was 40 ± 62.14 [*U* < 0.05, *p* < 0.001].

#### Multivariant Study of the Consumption of Opioids

Using a multiple linear regression analysis, we evaluated the independent variables potentially related to the dose of opioid [Table 4]. The anaesthetic technique used was the only explanatory variable since EG patients required a significantly lower dose of opioids to manage their postoperative pain. The model explained 90.5% of the variance regarding the dose of opioids consumed. [F (9,50) =63.757, *p* < 0.001; R^2^ = 0.905].

## 4. Discussion

The objective of this study was to evaluate the postoperative analgesic effect in an experimental group (EG) that underwent peripheral femoral and posterior nerve blocks compared to a control group (CG) that underwent a femoral nerve block and elastomeric pump infusion of opioids and NSAIDs after TKA surgery.

Pain management after knee arthroplasty remains a challenge, with more than half of patients experiencing moderate to severe pain [10]. Consequently, many patients are treated with NSAIDs and analgesics, including opioids, which can cause significant side effects, especially for the elderly [11]. Some systematic reviews and meta-analyses have been carried out to evaluate various analgesic interventions in TKA [8]. To date, there is still no consensus on a standard technique; its management protocol therefore varies. In 2019, almost 60% of the centres in Spain were not following a single standard protocol regarding analgesics used in TKA surgery. The most common was a combination of femoral nerve block and neuraxial anaesthesia along with an infusion of NSAIDs and opioids, which is the protocol we follow in our centre [12].

This study has shown that adding a tibial nerve block to the femoral nerve block achieved the same postoperative analgesic effect at rest when compared to those who underwent a femoral nerve block along with the infusion of NSAIDs and opioids using an elastomeric pump. However, with joint movement the mean of the NPRS values reported was lower in the EG throughout the period studied and was statistically significant at 48 h after surgery. Consequently, the EG required less rescue analgesia within 48 h after TKA surgery.

The innervation of the knee is complex since its posterior capsule is innervated by articular branches coming mainly from the obturator nerve, sciatic nerve, and, particularly, the articular branches of the tibial nerve [8,13]. Some authors have addressed this issue, as we have done, by applying different anaesthetic techniques, some of which involve blocking other nerves responsible for knee sensitivity together with the femoral nerve block. Domagalska et al. [12] conducted a review of different locoregional techniques and showed that the best analgesic effect was obtained by combining a periarticular injecton or iPACK with a peripheral nerve block, particularly the adductor canal block. Hasabo et al. [14] found no differences in pain control within 48 h after TKA surgery or in opioid consumption when comparing the femoral nerve block with the adductor canal block. In addition, the superiority of the adductor canal block has been seen in terms of preserving quadriceps strength. Other authors, such as Mou et al. [15] and Et et al. [16] aimed to reduce the quadriceps block and proposed the implementation of an iPACK block associated with a peripheral nerve block, such as an adductor canal block, which allowed them to improve the analgesia, reduce postoperative opioid consumption, and improve knee functionality.

The location of the tibial nerve varies according to BMI and thigh diameter, being deeper in the popliteal fossa in patients with higher BMI and thigh diameter. In our study, both parameters were similar in both groups [17].

The advantages of tourniquet use in knee arthroplasty include benefits for surgeons and patients, ranging from a bloodless operating site to reduced intervention time. Time under ischemia is crucial as it can cause damage to leg muscles that could interfere with the analgesic effect [18]. In our study, both groups had similar ischemia times.

In this study, two-thirds of the patients in the EG were did not require opioid intake and experienced no detriment in pain control. Similarly, Sinha et al., 2012 [8] demonstrated the same analgesic effect in the EG, which underwent a tibial nerve block combined with a femoral nerve block, compared to the CG, which underwent a block of the sciatic nerve combined with femoral nerve block. In addition, the absence of a peroneal nerve block allowed for faster rehabilitation in the EG.

A strength of the present study is that all TKA operations were carried out by the same team of surgeons and anaesthesiologists. Another strength is that the NPRS evaluation and the rescue analgesia were carried out by nurses who were not told which procedure had been performed on each patient. One limitation of our study is the small sample size. A second limitation is that the dose of ropivacaine and the total number of analgesics administered were different in both groups. Finally, the benefit of having reduced opioid consumption in the EG could not be reported.

The future of analgesia in knee replacement surgery lies in finding the best regional and intravenous techniques for each patient to achieve pain control, enabling rapid rehabilitation, reducing side effects, and shortening hospital stays. This study demonstrates that performing other peripheral nerve blocks in addition to the femoral nerve block can achieve pain control while reducing opioid consumption.

## 5. Conclusions

Adding an ultrasound-guided tibial nerve block to the femoral nerve block for total knee arthroplasty may achieve the same analgesic efficacy within 48 h after surgery as a femoral nerve block plus opioids and NSAIDs. The tibial nerve block could potentially reduce opioid use after TKA surgery.

## Figures and Tables

**Table 1:** Variables studied in the experimental and control groups at baseline.

Variable	Experimental Group	Control Group	Significance
Gender:			
-Men	15 [50%]	16 [53.3%]	Phi = 0.067, *p* = 0.796 ^1^
-Women	15 [50%]	14 [46.7%]	
Age [years]	69.60 ± 6.57	68.07 ± 7.65	*U* = 410, *p* = 0.553 ^2^
Barthel index	98.33 ± 3.55	98 ± 4.84	*U* = 442, *p* = 0.861 ^2^
ASA:			
-Type 1	0 [0%]	3 [10%]	
-Type 2	22 [73.3%]	25 [83.3%]	Phi = 0.336, *p* = 0.034 ^1^
-Type 3	8 [26.7%]	2 [6.7%]	
BMI	29.92 ± 3.58	29.98 ± 3.20	*U* = 446, *p* = 0.953 ^2^
Thigh diameter	44.68 ± 4.18	45.15 ± 1.36	*U* = 426.5, *p* = 0.727 ^2^
Haemoglobin [g/dL]	14.82 ± 1.17	14.52± 1.55	t = −0.922, *p* = 0.360 ^3^
eGFR [mL/min/1.73^2^]	86.03 ± 7.13	86.60 ± 6.04	*U* = 444, *p* = 0.922 ^2^

^1^, Chi-squared test; ^2^, Mann–Whitney *U* test; ^3^, Student’s *t*-test. ASA: American Society of Anaesthesiologists physical status classification system; BMI: body mass index; eGFR: estimated glomerular filtration rate. The two groups were homologous for number, sex, age, Barthel index, BMI, thigh diameter, preoperative haemoglobin, and eGFR. There were more patients with an ASA of 3 in the EG.

**Table 2:** Intraoperative variables studied in both groups.

Variable	Experimental Group	Control Group	Significance
Type of prosthesis			
-Innex	18 [48.6%]	19 [51.4%]	Phi = 0.034, *p* = 0.791 ^1^
-Gemini	12 [52.2%]	11 [47.8%]	
Tranexamic acid	26 [86.7%]	29 [96.7%]	Phi = 0.181, *p* = 0.161 ^1^
Time of surgery(min)	87.00 ± 12.70	84.30 ± 15.66	*U* = 395, *p* = 0.393 ^2^
Time of ischemia (min)	58.07 ± 13.11	55.67 ± 12.62	*U* = 410, *p* = 0.553 ^2^
Blood loss (mL)	116.67 ± 46.11	118.33 ± 44.49	*U* = 438, *p* = 0.805 ^2^

^1^, chi-squared test; ^2^, Mann–Whitney *U* test. The type of prosthesis implanted, the surgery duration, the time of ischemia and the blood loss were similar in both groups.

**Table 3:** Variables studied for rescue analgesia.

Independent Variable	Exp (B)	CI 95%	Significance
Anaesthetic technique	0.067	0.007–0.634	*p* = 0.018
BMI	1.081	0.845–1.383	*p* = 0.536
Surgery time	1.027	0.944–1.117	*p* = 0.533
Time of ischemia	1.008	0.929–1.095	*p* = 0.842
Bromage	1.001	0.985–1.017	*p* = 0.899
Type of prosthesis	0.330	0.061–1.796	*p* = 0.20
ASA	0.943	0.113–7.860	*p* = 0.957
Gender	1.082	0.189–6.200	*p* = 0.929
Age	1.080	0.944–1.235	*p* = 0.265
Barthel index	1.155	0.882–1.513	*p* = 0.296

The anaesthetic technique used determined the need for rescue analgesia within 48 h after surgery in the patients who underwent total knee arthroplasty.

**Table 4:** Variables studied for opioid consumption.

Variable	B	95% CI	Βeta	T	*p*
Anaesthesia technique	−256.895	[−279.4–(−234.38)]	−0.93	−22.92	<0.001
BMI	−4.758	[−9.21–(−0.30)]	−0.11	−2.14	0.097
Thigh diameter	3.976	[0.09–7.86]	0.11	2.05	0.055
Time of surgery	0.351	[−0.85–1.55]	0.03	0.58	0.56
Time of ischemia	−0.470	[−1.81–0.87]	−0.04	−0.70	0.48
Blood loss	0.246	[−0.021–0.51]	0.08	1.85	0.07
Bromage	0.042	[−0.179–0.26]	0.16	0.38	0.70
Age	0.171	[−1.54–1.88]	0.009	0.20	0.84
eGFR	1.443	[−0.50–3.38]	0.06	1.48	0.14

The anaesthetic technique was associated with the dose of opioids consumed. The experimental group consumed 256.8 milligrams less opioids after total knee arthroplasty.

## Data Availability

The database used and analysed is available from the corresponding author on research request.
